# Untangling Relational Ties: How Internalized Homonegativity and Adult Attachment Shape Relationship Quality in Lesbian and Gay Couples

**DOI:** 10.3390/bs15020205

**Published:** 2025-02-13

**Authors:** Tommaso Trombetta, Chiara Fusco, Luca Rollè, Alessandra Santona

**Affiliations:** 1 Department of Psychology, University of Turin, 10124 Turin, Italy; tommaso.trombetta@unito.it (T.T.); l.rolle@unito.it (L.R.); 2 Department of Psychology, University of Milano Bicocca, 20126 Milan, Italy; alessandra.santona@unimib.it

**Keywords:** internalized homonegativity, minority stress, adult attachment, negative affect, positive affect, couple relationship quality, dyadic adjustment, LGBTQ+

## Abstract

Introduction: Several studies point to an association between minority stress, especially internalized homonegativity and the quality of the couple relationship. However, the dimensions of psychological functioning that might moderate this relationship seem to be scarcely explored. Accordingly, the present study aimed to investigate the association between internalized homonegativity and relationship quality by examining the moderating role of adult attachment (dimensions of anxiety and avoidance) in lesbian and gay (LG) people. Materials and Methods: A total of 674 LG participants who had been in a couple relationship for at least 12 months were included in the study and completed self-report questionnaires. Results: The results show a direct negative effect of internalized homonegativity on relationship quality. Adult attachment seems to moderate this relationship: on the one hand, the negative relationship between internalized homonegativity and relationship quality is significant at high levels of anxious attachment, but not at low levels. On the other hand, the negative relationship between internalized homonegativity and relationship quality turns out to be significant at low levels of avoidant attachment, but not at high levels. Discussion: The results suggest that attachment anxiety and avoidance play different roles in the relationship between internalized homonegativity and relationship quality. While high levels of anxious attachment appear to be a risk factor, high levels of avoidant attachment seem to play a protective role. Although future studies are needed to investigate the present preliminary findings further, the results of the present study provide useful clinical and research insights.

## 1. Introduction

Minority stress refers to the unique and specific stress conditions faced by LGBTQ+ people—specifically including lesbian and gay (LG) people—due to heterosexist and discriminatory attitudes directed against them because of their sexual orientation (Meyer, 1995, 2003). The minority stress model formulated by Meyer (1995, 2003) describes three main stressors that lie on a continuum from the most distal to the most proximal. These include experiences of discrimination—i.e., experiences of aggression and microaggression directed at LG individuals because of their sexual orientation (distal stressor)—, perceived stigma—i.e., the perception of being stigmatized and discriminated against because of one’s sexual orientation (proximal stressor)—, and internalized homonegativity. Internalized homonegativity (proximal stressor) refers to negative attitudes and affect directed at one’s self and sexual orientation because of the internalization of social homophobic attitudes (Meyer, 2003). These include negative global attitudes towards homosexuality, disengagement from the LGBT+ community, and discomfort with disclosure of sexual orientation and same-sex sexual behaviors. This internalization can lead to psychological conflicts that can affect self-esteem and well-being in general (Meyer & Dean, 1998; Newcomb & Mustanski, 2010).

The minority stress model has proven to be fundamental to understanding the factors that may negatively impact the well-being of LG individuals, and a large body of research (Baiocco et al., 2023; Dürrbaum & Sattler, 2020; Frost & Meyer, 2023; Hoy-Ellis, 2023) over the past decades has demonstrated its validity and predictive power.

Although the minority stress model was originally applied primarily to examine the antecedents of individual well-being in LG people (e.g., in terms of anxiety, depression, and suicide risk), it has been suggested that minority stressors may be a source of relational stress for LG individuals (Meyer & Frost, 2013), and several studies have demonstrated the negative effects of minority stress on couple relationship quality (Bresin et al., 2023; Doyle & Molix, 2015; Rostosky & Riggle, 2017; Song et al., 2023). Specifically, discrimination and stigma at multiple levels (structural, social, and psychological) can influence the recognition of LG couples by also affecting how they perceive the quality of their own relationship, partly due to the internalization of this discrimination. For example, the lack of legal recognition of LG marriages in some countries, such as Italy, as well as workplace discrimination that limits the sharing of relationship experiences, and internalized negative attitudes that tend to promote negative representations of same-gender relationships (e.g., in terms of legitimacy, stability, trust, and satisfaction), can negatively impact the quality of couple relationships and their functioning (Rostosky & Riggle, 2017). This appears to be reflected in data from several studies showing an association between minority stress and various indicators of couple relationship quality, including quality of communication (Scott et al., 2023), intimate partner violence (Balsam & Szymanski, 2005; Li et al., 2021; Trombetta et al., 2023; Trombetta & Rollè, 2022), relationship satisfaction (Sommantico et al., 2019), dyadic adjustment (Balsam & Szymanski, 2005; Scott et al., 2023; Song et al., 2022; Szymanski & Hilton, 2013), and general indexes of relationship quality (Feinstein et al., 2018; Li et al., 2021). The present study fits into this line of research by assessing the relationship between internalized homonegativity and relationship quality and examining the moderating role of adult attachment (anxiety and avoidance).

In the broader psychological literature, the terms “relationship quality”, “relationship satisfaction”, and “couple adjustment” are frequently used interchangeably and broadly defined as a multifaceted concept that refers to how positively or negatively individuals feel about and adjust to their intimate relationships with a partner (Delatorre & Wagner, 2020; Morry et al., 2010). Specifically, the quality of the couple’s relationship has been assessed by scholars by evaluating patterns of interaction between partners and their internal representations of the relationship (Farooqi, 2014; Acitelli, 2008), with dyadic adjustment being defined as a multidimensional construct that serves as an indicator of relationship quality, reflecting the relational balance achieved by a couple within a process that evolves throughout the entire life cycle (Spanier & Cole, 1975; Spanier, 1976; Garbarini, 2011; Garbarini et al., 2014).

Several studies have supported the hypothesis that the internalization of stigma has a specific influence on relationship quality (Feinstein et al., 2018; Frost & Meyer, 2009; Szymanski & Hilton, 2013). Two recent meta-analyses (Doyle & Molix, 2015; Song et al., 2023) have demonstrated a significant negative effect of minority stress on the couple relationship functioning, with the effect size being greater for internalized stigma than for perceived stigma.

While the adverse effects of minority stress on couple relationship quality have been confirmed in several studies, other studies (Kamen et al., 2011; Scott et al., 2023) demonstrated inconsistent results that could be due to moderating factors and other methodological limitations (e.g., small sample size; Doyle & Molix, 2015). Accordingly, recent studies have examined the factors and dimensions of psychological functioning that may modulate the negative effects of minority stress on individual and relational well-being of LG people. Investigating and deepening the role of these dimensions is essential to provide useful guidance for developing and implementing research and interventions to promote health in the LG population and counteract the negative effects of discrimination and internalization of stigma. However, most studies have focused on the variables that moderate the effect of minority stress on individual well-being, while a few studies (J. J. Mohr et al., 2013; Rosenthal & Starks, 2015; Sullivan et al., 2017) have specifically looked at couple relationship quality, and further studies are needed.

In this context, adult attachment could be seen as a factor that may moderate the effects of minority stress, and internalized homonegativity in particular, on couple relationship quality (J. Mohr, 2008). Building on Bowlby’s attachment theory (Bowlby, 1969, 1973, 1980), scholars highlighted how attachment patterns shape adult relationships, guiding emotional and behavioral regulation in close relationships (Mikulincer & Shaver, 2016; Shaver et al., 2016). Adult attachment refers to the intimate bond between partners that promotes physical closeness and emotion regulation in times of distress, with romantic partners being conceptualized as reciprocal attachment figures (Hazan & Shaver, 1987). The development of secure attachment and mature emotion regulation skills is promoted by relationships with available and responsive attachment figures. Conversely, unavailable and unresponsive attachment figures undermine the development of secure attachment and promote insecure attachment and secondary strategies of regulation of the attachment system (strategies of hyperactivation and deactivation of the attachment system) that, while adaptive to the environment in which they develop, may later malfunction and be detrimental to individual and relational well-being (Mikulincer et al., 2003; Mikulincer & Shaver, 2005, 2016). According to Mikulincer and Shaver (2005, 2007a), the functioning of the attachment system and the secondary emotion regulation strategies can be assessed on the basis of two dimensions: attachment anxiety and avoidance. On the one hand, higher levels of attachment anxiety imply separation anxiety and vulnerability to perceived abandonment cues, accompanied by a tendency to hyperactivate the attachment system, resulting in high reactivity to frustration and the intense expression of attachment needs in order to achieve the desired closeness to the partner. On the other hand, individuals with a higher level of attachment avoidance are characterized by discomfort with closeness and fear of intimacy. They tend to deactivate the attachment system to promote self-reliance and interpersonal and emotional distance from the partner, as well as limit access to negative affect and attachment needs in order to prevent the occurrence of frustration and fear of rejection. While these strategies originate from early attachment patterns formed in childhood, they persist into adulthood, influencing how people cope with closeness and distance in romantic and peer relationships (Mikulincer & Shaver, 2016; Simpson, 1990).

While the development of secure attachment can be a protective factor that can promote mature emotional regulation in situations of distress and frustration, insecure attachment and reliance on secondary strategies to regulate the attachment system can negatively impact individual and relational well-being and exacerbate the negative effect given by adverse conditions and distress (Mikulincer & Shaver, 2007a, 2007b). Accordingly, several studies emphasized the moderating role that adult attachment plays in the relationship between adverse and stressful conditions—e.g., depression (Heene et al., 2005), solitude (S. Zhang & Li, 2021), cyber dating abuse (Lancaster et al., 2020), living with ADHD children (Sochos & Yahya, 2015), social disapproval of the relationship (Bradford et al., 2020)—and the quality of the couple relationship. These data show that insecure attachment tends to increase the impact of stressful conditions on the quality of the couple relationship, whereas secure attachment appears to be a protective factor (Heene et al., 2005).

Although some studies (Lancaster et al., 2020; Sochos & Yahya, 2015; Vigl et al., 2022) suggest that higher levels of attachment anxiety and avoidance are both risk factors for couple relationship quality and increase the negative effects of adverse conditions, other studies (Bar-Shachar et al., 2022; Itzhaky et al., 2017) show different results depending on the attachment dimension considered. For example, Bar-Shachar et al. (2022) found that high attachment anxiety increased the impact of perceived support from the partner in response to COVID-19-related concerns on relationship satisfaction, while attachment avoidance seems to buffer the influence of negative partner’s behaviors in response to COVID-19-related concerns on relationship satisfaction, acting as a protective factor. Similarly, a recent study (Itzhaky et al., 2017) involving 504 Lebanon war veterans found that although the interaction between Posttraumatic Stress Symptoms (PTSS) and attachment avoidance was not significant (although it approached significance), the data showed that at low levels of attachment avoidance there was evidence of a negative effect of PTSS on relationship quality, whereas at high levels of attachment avoidance the relationship between PTSS and relationship quality was not significant, again indicating the protective role of attachment avoidance. In turn, at high levels of attachment anxiety there was a negative relationship between PTSS and relationship quality, whereas at low levels of attachment anxiety this relationship was not significant (the interaction between PTSS and attachment anxiety was statistically significant). However, several other studies pointed to the direct negative impact of avoidance on individual and couple well-being (Cataudella et al., 2023; Tognasso et al., 2022; Trombetta et al., 2024; X. Zhang et al., 2022; Zheng et al., 2020). Although strategies of deactivation of the attachment system may help to limit access to attachment needs and negative affect (for defensive purposes), high levels of stress, limited psychological resources, and negative self-representations (Zakalik & Wei, 2006) may reduce the effectiveness of such defensive strategies and promote the emergence of negative perceptions and affect (Mikulincer & Shaver, 2014). Moreover, all the studies that had previously examined the moderating role of adult attachment on the association between stressful conditions and couple relationship quality included mainly heterosexual participants. In contrast, there appear to be few studies that have examined these aspects in gay and lesbian individuals. To our knowledge, only one previous study (J. J. Mohr et al., 2013) has examined the relationship between minority stress and relationship quality. However, the data obtained do not suggest a moderating effect in this relationship given by adult attachment. Given the partially contradictory results regarding the role of adult attachment (especially attachment avoidance) and the few studies conducted on LG individuals to examine the factors that may modulate the effects of minority stress on couple relationship quality, further studies in this direction are needed.

Accordingly, the present study aims to further investigate the factors that may modulate the influence of internalized homonegativity on the quality of the couple relationship by examining the moderating effect of adult attachment (anxiety and avoidance dimensions) in the relationship between the two variables. The results of the study may provide useful insights at the clinical level by highlighting the dimensions of psychological functioning that should be targeted for intervention to counteract the negative effects of internalized homonegativity on the couple’s well-being. Furthermore, these findings could have important research implications by informing future studies on the interplay between attachment and internalized homonegativity, guiding the identification of key mechanisms that influence relationship quality in LG couples. Considering the relationship between couples’ well-being and individual health, these data gain further importance, especially in the LGBT+ population, where the discriminatory and heterosexist context may be a barrier to accessing competent and inclusive services (Santoniccolo et al., 2021).

### Hypotheses

**H1:**  *Internalized homonegativity is negatively associated with relationship quality.*

**H2:**  *Attachment anxiety moderates the association between internalized homonegativity and relationship quality: at high levels of attachment anxiety, the negative relationship between internalized homonegativity and relationship quality would be statistically significant*.

Given the conflicting data on attachment avoidance, we intend to take an exploratory approach to its moderating role in the relationship between internalized homonegativity and relationship quality.

## 2. Materials and Methods

### 2.1. Procedure

We recruited participants through calls posted on different social media (e.g., Facebook and Instagram) and the collaboration of LGBT+ associations, which helped disseminate the research project. No compensation was offered for taking part in the study.

We followed the provisions of Italian law 196/2003 and collected participants’ informed consent before they started fulfilling the questionnaire. The Ethics Committee of the Psychology Department of the University of Milano Bicocca had previously approved the research project (protocol number: RM-2020-276, 8 April 2020). When accessing the online research protocol on the Qualtrics platform, participants read a brief explanation about the content and purpose of the study, then they were administered the self-report questionnaires described below.

### 2.2. Participants

We excluded from the study those participants who did not complete the research protocol, were not in a same-gender relationship, were under 18 years of age, or identified with a sexual orientation other than gay or lesbian. Additionally, only participants who reported that their current relationship had lasted for at least one year were considered.

The present study involved 674 participants, including 237 people identifying as men (34.7%) and 437 people identifying as women (65.3%). Participants’ ages varied between 18 and 70 years (mean age: 33.73 years; SD = 9.4). Additional sociodemographic information is reported in Table 1.

Sexual orientation was measured using the Kinsey scale (Kinsey et al., 1948), with participants reporting the following answers in relation to their sexual orientation: no participants declared to be exclusively heterosexual; 8 participants reported being predominantly heterosexual, sometimes homosexual (1.2%); 9 reported being predominantly heterosexual, but with a strong homosexual component (1.3%); 52 participants referred to identifying both as heterosexual and homosexual, equally (7.7%); 43 reported being predominantly homosexual, but with a strong heterosexual component (6.4%); 190 participants stated they were predominantly homosexual, but in some circumstances heterosexual (28.2%); finally, 372 reported identifying exclusively as homosexual (55.2%).

### 2.3. Measures

Participants first completed a survey that included the collection of sociodemographic information and questions about their intimate relationships. Then, they were asked to complete the following measures, which were presented in the research protocol as they are listed in the present paper.

### 2.4. Internalized Homonegativity

The Measure of Internalized Sexual Stigma for Lesbians and Gay Men (MISS-LG; Lingiardi et al., 2012) is a self-report questionnaire that examines internalized homonegativity across three dimensions, categorized into three subscales: the *Identity subscale*, the *Social Discomfort subscale*, and the *Sexuality subscale*. The MISS-LG consists of 17 items on a 5-point Likert scale (from 1 = ‘totally disagree’ to 5 = ‘totally agree’). The Identity subscale (5 items) indicates an enduring propensity to have a negative self-attitude and to consider sexual stigma as a part of self-identity (e.g., “If it were possible, I would do anything to change my sexual orientation”). The Social Discomfort subscale (7 items) reflects fear of public identification as a lesbian woman or gay man in the social context and disclosure in private and professional life (e.g., “When I feel attracted to another lesbian/gay man, I hope no one realizes it”). The Sexuality subscale (5 items) indicates the pessimistic attitudes toward the quality and duration of intimate relationships in lesbians and gay men (e.g., “Lesbians/gay men can only have flings/one-night stands”) and the negative conception of gay or lesbian sexual behaviors (e.g., “When I have sex with a woman/man, I feel awkward”). Two versions of the instruments were developed to account for gay and lesbian people. Both versions share 11 identical items, which show the strongest correlations with the same factors across both groups. However, six items differ to account for gender-specific experiences. For instance, the gay men’s version includes the item, “If you are gay, it’s better to have an ‘active’ sexual role,” while the lesbian version features, “Even if I am attracted to women, I think that a woman who has had sexual experiences with men is more complete.” Higher scores on this instrument are related to greater levels of internalized homonegativity. The overall instrument showed high internal consistency (Cronbach’s α = 0.87), with the subscales showing moderate to high internal consistency (respectively, Cronbach’s α = 0.83 for the Identity subscale, Cronbach’s α = 0.79 for the Social Discomfort subscale, and Cronbach’s α = 0.61 for the Sexual subscale).

### 2.5. Relationship Quality

The Dyadic Adjustment Scale (DAS; Spanier, 1976; Italian validation by Gentili et al., 2002) was administered to assess the couple’s relationship quality, defined through the multidimensional construct of dyadic adjustment of partners within their couple relationship at a given time. It consists of 32 items divided into four subscales, each one representing one of the interrelated dimensions composing dyadic adjustment: 1. Dyadic Consensus (13 items), i.e., the consensus between the couple members on important issues and decisions (e.g., “Aims, goals, and things believed important”); 2. Dyadic Cohesion (5 items), i.e., the sharing of activities and interests between the partners (e.g., “Do you and your mate engage in outside interests together?”); 3. Dyadic Satisfaction (10 items), i.e., the satisfaction referred to the current state of the relationship (e.g., “How often do you discuss or have you considered divorce, separation or terminating your relationship?”); and 4. Affective Expression (4 items), i.e., the satisfaction referred to sexual and emotional life within the couple’s relationship (e.g., “Sex relations”). The overall score of Dyadic adjustment ranged from 0 to 151, with higher scores indicating well-adjusted relationships. The internal consistency of the total score was high (Cronbach’s α = 0.89), and subscales showed moderate to high internal consistency (resulting in Cronbach’s α = 0.87 for the Dyadic Consensus subscale, Cronbach’s α = 0.71 for the Dyadic Cohesion subscale, Cronbach’s α = 0.77 for the Dyadic Satisfaction subscale, and Cronbach’s α = 0.62 for the Affective Expression subscale).

### 2.6. Adult Attachment

To assess adult attachment anxiety and avoidance, we administered the *Experiences in Close Relationships-Revised* (ECR-R; Fraley et al., 2000; Italian validation by Busonera et al., 2014). The ECR-R is a self-report questionnaire consisting of 36 items, which assesses adult attachment based on two key dimensions: attachment anxiety and attachment avoidance. The ECR-R comprises two 18-item subscales, respectively measuring attachment anxiety and avoidance. Respondents are asked to rate each item on a seven-point Likert scale going from 1 (‘strongly disagree’) to 7 (‘strongly agree’), with higher scores reflecting higher levels of attachment anxiety and avoidance. The attachment anxiety subscale measures the respondent’s level of concern regarding abandonment and rejection in intimate relationships and the use of strategies of hyperactivation of the attachment system (e.g., “I often worry that my partner will not want to stay with me”). The attachment avoidance subscale measures the respondent’s level of discomfort with closeness and dependency in close relationships and the use of strategies of deactivation of the attachment system (e.g., “I don’t feel comfortable opening up to romantic partners”). In the present study, internal consistency for attachment anxiety and avoidance subscales was high (Cronbach’s α = 0.87 and 0.83, respectively).

### 2.7. Analysis Plan

Analyses were performed with R (R Development Core Team, 2021). First, descriptive statistics and Pearson’s bivariate correlations were computed for all continuous variables in the study. In addition, *t*-tests were conducted to examine the role of gender in its association with the study variables, providing further insight into potential gender-based differences. Second, to explore the moderation model, a multiple linear regression model was used to test the effects of internalized homonegativity, attachment anxiety and avoidance, and their interaction on dyadic adjustment. If a significant interaction term was found, a simple slope analysis was performed, and data were plotted. In addition, we considered participants’ age, gender, relationship duration, and whether they were childless or had children as covariates in the model.

## 3. Results

### 3.1. Correlations

Correlation analyses showed that attachment avoidance (r = 0.256, *p* < 0.001) and anxiety (r = 0.224, *p* < 0.001) positively correlated with internalized homonegativity, suggesting that high levels of attachment avoidance and anxiety are associated with high levels of internalized homonegativity (see Table 2).

Dyadic adjustment showed a negative statistically significant correlation with both attachment avoidance (r = −0.525, *p* < 0.001) and attachment anxiety (r = −0.363, *p* < 0.001), displaying that high levels of attachment anxiety or avoidance seem to be related to lower levels of dyadic adjustment. Finally, dyadic adjustment was also negatively statistically significantly correlated with internalized homonegativity (r = −0.210, *p* < 0.001), showing that lower levels of dyadic adjustment are associated with higher levels of internalized homonegativity.

### 3.2. Gender Differences in Levels of Internalized Homonegativity, Adult Attachment Dimensions, and Dyadic Adjustment

An independent-sample *t*-test was carried out to test whether participants’ gender statistically significantly affected the variables considered in this study (see Table 3). Results showed that internalized homonegativity and attachment avoidance seem to statistically significantly differ based on participants’ gender. Specifically, we found a significant difference in attachment avoidance and internalized homonegativity between men and women, with men reporting significantly higher mean scores than women.

### 3.3. The Moderating Role of Attachment Dimensions in the Relation Between Internalized Homonegativity and Dyadic Adjustment

We computed a multiple linear regression model to investigate the main effects of internalized homonegativity and attachment dimensions on dyadic adjustment and the moderating effect of attachment dimensions on the relation between internalized homonegativity and adjustment. In addition, we controlled the effects for participants’ age, gender, relationship duration, and whether they had children. We employed a bootstrap method with 1000 iterations to estimate the regression parameters (Peng, 2024). The resulting distributions of these parameters are presented in the Supplementary Materials, providing additional evidence of the robustness of the model. The full model explained 37% of the variance in dyadic adjustment (R^2^ = 0.37, adjusted R^2^ = 0.358, F(9,664) = 42.60, *p* < 0.001), with a residual standard error of 12.80.

The model showed the statistically significant negative main effects of internalized homonegativity (β = −0.159, SE = 0.07, t(664) = −2.43, *p* = 0.015), attachment anxiety (β = −0.156, SE = 0.03, t(664) = −5.26, *p* < 0.001), and attachment avoidance (β = −0.528, SE = 0.04, t(664) = −13.11, *p* < 0.001) on dyadic adjustment. Additionally, the analysis showed significant interaction effects between internalized homonegativity and attachment anxiety (β = −0.010, SE = 0.00, t(664) = −3.00, *p* = 0.003), as well as between internalized homonegativity and attachment avoidance (β = 0.009, SE = 0.00, t(664) = 2.14, *p* = 0.036), indicating that the relationship between internalized homonegativity and dyadic adjustment scores varies according to the levels of the attachment dimensions. We did not find any significant differences controlling for participants’ gender, and relationship duration, while we observed statistically significant differences in relation to participants’ age and between those who reported being childless and those with children. Specifically, age showed a positive and statistically significant effect on dyadic adjustment (β = 0.144, SE = 0.07, t(664) = 2.17, *p* = 0.030), suggesting that, with increasing age, there may be a slight enhancement in dyadic adjustment. Finally, the presence of children was statistically significantly associated with lower levels of dyadic adjustment (β = −7.203, SE = 1.17, t(664) = −6.13, *p* < 0.001).

**Figure 1:**
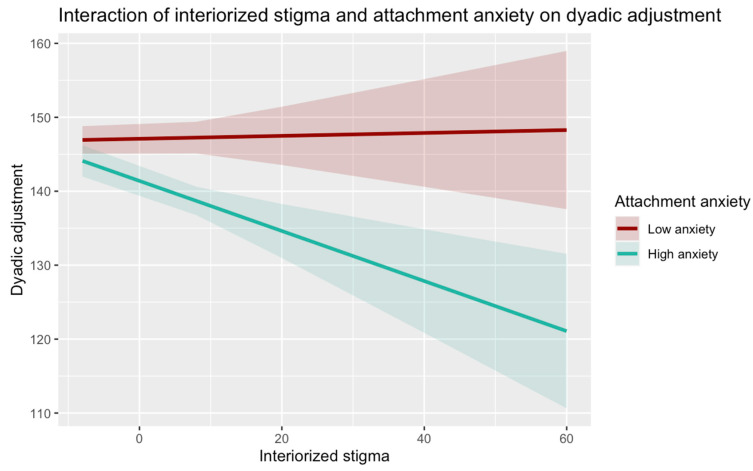
Interaction of internalized homonegativity and attachment anxiety on dyadic adjustment.

**Figure 2:**
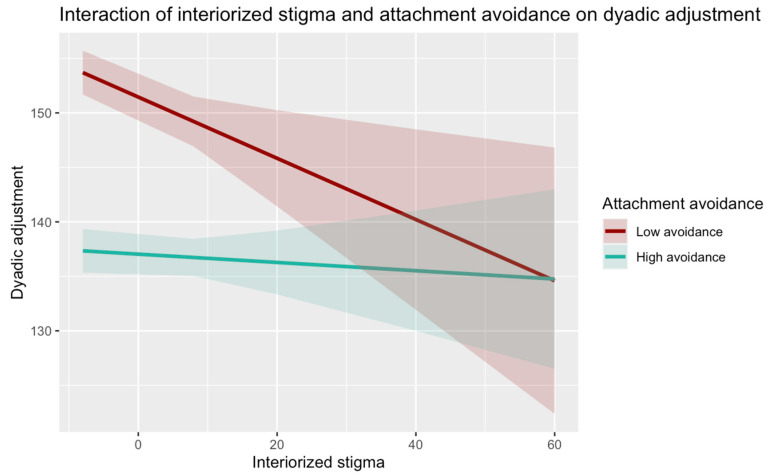
Interaction of internalized homonegativity and attachment avoidance on dyadic adjustment.

We then performed a simple slope analysis to understand the meaning of the statistically significant interaction effects. Figure 1 and Figure 2 show the simple slope analyses to interpret the moderation effect. The interaction between internalized homonegativity and attachment anxiety was statistically significant: specifically, at high levels of attachment anxiety, internalized homonegativity was negatively associated with dyadic adjustment (β = −0.035, SE = 0.09, t(664) = −3.95, *p* < 0.000), while at low levels of attachment anxiety, internalized homonegativity did not show a significant effect on dyadic adjustment (β = 0.031, SE = 0.09, t(664) = 0.35, *p* = 0.725). Additionally, the interaction between internalized homonegativity and attachment avoidance was statistically significant too: at low levels of attachment avoidance, internalized homonegativity was negatively associated with dyadic adjustment (β = −0.285, SE = 0.10, t(664) = −2.81, *p* = 0.005); in turn, at high levels of attachment avoidance, internalized homonegativity did not show a significant effect on dyadic adjustment (β = −0.037, SE = 0.07, t(664) = −0.515, *p* = 0.607).

## 4. Discussion

The aim of the present study was to assess the impact of internalized homonegativity on the quality of couple relationships by examining the moderating role of adult attachment (anxiety and avoidance). This study is part of an emerging strand of research that focuses on assessing the factors that may modulate the negative effects of minority stress on individual and couple well-being in order to provide useful insights for research and interventions to promote the health of LGBTQ+ individuals.

Consistent with H1, study’s findings showed a negative association between internalized homonegativity and couple relationship quality. The results are in line with previous literature (Doyle & Molix, 2015; Feinstein et al., 2018; Frost & Meyer, 2009; Song et al., 2023; Szymanski & Hilton, 2013) and confirm the spillover effect of minority stress on couple’s relationship. Individuals with higher levels of internalized homonegativity may develop negative representations about themselves and one’s own personal sexual desires and behaviors, their partners, and same-gender relationships in general, as a consequence of the internalization of socially expressed homonegative and heteronormative attitudes that delegitimize and discriminate against the sexuality and intimate relationships of LG individuals. This may contribute to negative affect and perceptions regarding the couple relationship that might reduce its perceived quality. Moreover, as suggested by other authors (Brown, 1994; Szymanski & Hilton, 2013; Zevy & Cavallaro, 1987), higher levels of internalized homonegativity may limit the ability to comprehend and express one’s feelings and emotions (also for defensive purposes), as also shown by various studies indicating an association between internalized homonegativity and emotion dysregulation (Lewis et al., 2014; Mann et al., 2022; Pachankis et al., 2015; Rendina et al., 2017; Szymanski et al., 2014; Trombetta et al., 2023; Trombetta & Rollè, 2023), with possible effects on communication, conflict, and intimacy within the couple relationship.

Considering the results of the moderation analyses (H2), it appears that the two dimensions of adult attachment influence the effect of internalized homonegativity on relationship quality differently. While attachment anxiety appears to be a risk factor, attachment avoidance seems to play a protective role. Indeed, the results show that, on the one hand, at high levels of attachment anxiety a significant negative effect of internalized homonegativity on relationship quality was found, while at low levels of attachment anxiety this association is not significant. These results may be explained by the fact that individuals with higher levels of attachment anxiety tend to hyperactivate the attachment system, showing increased vulnerability to attachment needs and negative affect (Mikulincer et al., 2003; Mikulincer & Shaver, 2005; Shaver & Mikulincer, 2002) that seems to exacerbate the detrimental effects of internalized homonegativity on relationship quality. This could also be related to the intense and sometimes dysregulated expression of attachment needs that is typical of people with high levels of attachment anxiety. This could undermine the quality of communication and increase conflict (J. J. Mohr et al., 2013), depriving the individual of the protective effect of positive relationships that may contribute to the co-regulation of distress and frustration associated with the experience of minority stress. In addition, hypervigilance to signs of abandonment and separation may lead these individuals to perceive their partners as unresponsive and untrustworthy (Mikulincer & Shaver, 2008, 2019), exacerbating the effects of internalized homonegativity on relationship quality. In contrast, low levels of attachment anxiety seem to represent a protective factor that can reduce the negative effects of internalizing homonegative attitudes on relationship quality, promoting more secure strategies of regulation and expression of the negative affect and perceptions due to minority stress. This may limit the spillover effect of internalized homonegativity on the couple relationship quality.

On the other hand, the results show that internalized homonegativity has a significant negative effect on relationship quality at low levels of attachment avoidance, whereas at high levels of attachment avoidance the association between internalized homonegativity and relationship quality is not statistically significant. The protective effect given by high levels of attachment avoidance could be due to the related strategies of deactivation of the attachment system. These strategies could indeed limit access to attachment needs and negative perceptions and affect for defensive purposes, thus reducing the perceived impact on relationship quality given by stressful conditions such as high levels of internalized homonegativity or other minority stressors (Mikulincer et al., 2003; Zakalik & Wei, 2006). However, these results may be at least partially related to the level of internalized homonegativity, which was relatively low in the participants of the present study. Indeed, it has been pointed out that while deactivation strategies may be adaptive in the context in which they develop, they may become ineffective and non-adaptive under later conditions characterized by high distress and frustration (Mikulincer & Shaver, 2014; Mikulincer et al., 2003). In these conditions, individual’s deactivation strategies and resources may not be sufficient to limit access to attachment needs, negative cognitions, and emotions. This may leave them exposed to higher levels of distress in the absence of secure emotion regulation strategies and a supportive social network, which can also be due to the fear of intimacy, related to the fear of rejection that characterizes individuals with high levels of attachment avoidance. This hypothesis seems to be supported by several studies (Cataudella et al., 2023; Tognasso et al., 2022; Trombetta et al., 2024; X. Zhang et al., 2022; Zheng et al., 2020), showing that higher levels of attachment avoidance negatively affect the well-being of individuals and couples. A comparison between populations with high levels of homophobia (e.g., clinical groups) and low-level groups (e.g., high- vs. low-risk samples) could further clarify the data obtained in this study and the role of attachment avoidance in the interplay between internalized stigma and couples’ functioning.

Interestingly, in the present study, low levels of attachment avoidance appear to be a risk factor that increase the negative impact of internalized homonegativity on the quality of the couple relationship. This finding seems to suggest that the lack of deactivation strategies of the attachment system may contribute to LG individuals’ vulnerability to the effects of internalizing discriminatory and homonegative attitudes, depriving them of defensive strategies that could be useful to reduce access to negative and distressing mental contents related to minority stress and the stigmatizing and heteronormative context in which they live. However, these hypotheses are only preliminary and partly speculative. Future studies should not only replicate this model to test its validity, but also investigate at which levels of internalized homonegativity deactivation strategies may constitute an adaptive defense and when they instead entail negative consequences at the individual and relational levels, as already highlighted in relation to the protective role of high levels of attachment avoidance.

Finally, while the results of the present study at least partially support the hypothesis (J. Mohr, 2008) that adult attachment may buffer the negative effects of minority stress on relationship quality (albeit in directions that were not hypothesized according to the data found in the present study on attachment avoidance), these findings are at odds with the sparse data in the literature showing no moderating effect of attachment anxiety and avoidance in the relationship between minority stress and relationship functioning (J. J. Mohr et al., 2013). Other studies are therefore needed to investigate these hypotheses further.

Regarding the clinical implications of the results found in the present study, adult attachment functioning and strategies to regulate interpersonal distance and affect could be mechanisms that interventions and clinicians should target to help these couples overcome the experiences of stigmatization and internalization of homophobia and the associated negative attitudes and affect. In this sense, attachment-informed interventions could help mitigate the negative effects of internalized homonegativity on relationship quality. Specifically, the data seem to suggest that for individuals with high levels of attachment anxiety, reducing reliance on strategies of hyperactivation of the attachment system and promoting secure-based strategies to regulate the mental states related to internalizing stigma and homophobia could be helpful in reducing stress levels and their impact on couple relationship quality. Attachment-informed interventions could be useful in this direction.

On the other hand, the results of the present study seem to indicate that the use and thus the promotion of attachment deactivation strategies could reduce the negative effects of internalized homonegativity on relationship quality. Hower, also in view of the exploratory nature of the present study, this should be treated with great caution. Indeed, it is also possible that attachment deactivation strategies are functional only in certain contexts and only for certain populations (e.g., populations at low risk in terms of stress exposure or with extreme levels of attachment avoidance), while becoming maladaptive in others. Indeed, several studies (Cataudella et al., 2023; Trombetta et al., 2024; X. Zhang et al., 2022) have highlighted the negative effects of attachment avoidance and related deactivating strategies on well-being, suggesting that while such strategies may be functional in the context in which they develop (e.g., in stigmatizing and rejecting environments), they may become dysfunctional in other situations and moments of life (e.g., in adult intimate relationships), leading to an exacerbation of the negative effects of stressful conditions (e.g., minority stressors). In this sense, other studies are needed to further investigate the role of attachment avoidance in the relationship between minority stress and couple (as well as individual) well-being and to provide clear clinical guidance.

## 5. Limitations and Future Directions

The results of the present study must be considered in light of some limitations. First, the study has a cross-sectional design. This does not allow clear and definitive conclusions to be drawn about the causal direction of the relationships found, although the hypotheses put forward are based on solid theoretical backgrounds such as attachment theory and the minority stress model. Future studies should, therefore, use a longitudinal design to test the direction of the relationships hypothesized here.

Second, the use of self-report instruments, as beneficial as they may be in terms of ease of administration and resources required, may have led to response bias related to, for example, social desirability. 

Third, only cisgender gay and lesbian people were included, with an imbalance in female gender. In addition, gay and lesbian people were considered as a single population, and separate analyses by gender were not conducted. Although gender was included as a control variable and no association with relationship quality was found, further studies are needed that include other sexual identities (e.g., bisexual, pansexual, transgender, and non-binary people) and conduct separate analyses by sexual orientation and gender identity.

Fourth, the participants involved in the present study were mainly white, highly educated, and with a good socioeconomic status. This can have influenced the results emerged, also in terms of generalizability. Future studies should replicate this model in other populations such as ethnic minorities or other marginalized groups.

Fifth, of the minority stressors included in Meyer’s model (Meyer, 1995, 2003), only internalized homonegativity was examined. Assessing the complex relationship between relationship quality, adult attachment, and other minority stressors, such as experiences of discrimination and perceived stigma, seems to be necessary to further examine the findings of the present study and to clarify the impact of multiple sources of minority stress on relationship quality.

Finally, it should be noted that data on the moderators of the relationship between minority stress and relationship quality are limited in the current literature. Further studies are needed to provide useful insights for the development and implementation of interventions to promote the health of LG individuals. Regarding the specific role of adult attachment, future studies seem necessary to investigate in particular the influence of attachment avoidance. In this direction, a comparison of groups exposed to high levels of distress with individuals exposed to low levels of distress could deepen the preliminary results found here and clarify the role of attachment avoidance and adult attachment more generally.

## 6. Conclusions

The results found in the present study show the moderating effect of attachment anxiety and avoidance in the relationship between internalized homonegativity and the quality of the couple relationship. The study suggests that the two dimensions of adult attachment play different roles in modulating the negative effects of internalized homonegativity on relationship quality. While high levels of attachment anxiety appear to be a risk factor for the couple’s well-being, high levels of attachment avoidance seem to play a protective role. Although other studies are needed to further investigate the findings of the present research (particularly in relation to the role of attachment avoidance), these preliminary results can provide a first insight into the usefulness of attachment-informed interventions aimed at promoting mature and secure strategies of emotional regulation.

This appears to be particularly relevant for individuals with high levels of attachment anxiety, whereas the results of the present study seem to suggest that the use of attachment system deactivation strategies, typical of higher levels of attachment avoidance, may be adaptive in reducing the negative effects of minority stress. However, these data should be taken with caution as they diverge from several studies showing negative effects on well-being due to high levels of attachment avoidance (Cataudella et al., 2023; Trombetta et al., 2024; X. Zhang et al., 2022). This may be linked to the levels of distress experienced, as high distress could render deactivation strategies ineffective, thereby increasing vulnerability (Mikulincer & Shaver, 2014). Future studies are therefore needed to investigate the role of attachment avoidance further and provide clear guidance for treatment.

Given the reciprocal influence between couple and individual well-being, analyzing the impact of minority stress on the quality of the couple relationship and the role of possible moderating variables gains additional importance. Indeed, the couple itself may be a resource to counteract the negative effects of discrimination and homophobia. Exploring variables that can strengthen relationship quality and reduce the impact of minority stress is therefore necessary in order to promote lesbian and gay people’s well-being.

## Figures and Tables

**Table 1:** Socio-demographic variables (N = 674).

	N	%
Educational level		
Middle school	13	1.9
High school diploma	241	35.8
Bachelor’s degree	347	51.5
Master’s degree, PhD	73	10.8
Duration of the relationship		
<2 years	169	25.1
2–5 years	302	44.8
5–10 years	116	17.2
10–15 years	49	7.3
>15 years	38	5.6
Relationship status		
Monogamous relationship	606	89.9
Open relationship	43	6.4
Other	25	3.7
Children		
Yes, mine	10	1.5
Yes, my partner’s	120	17.8
Yes, mine and my partner’s	28	4.2
No	510	75.7
Other	6	0.9
Children from previous relationships		
Yes	3	0.4
No	671	99.6
Financial status		
Financially precarious	79	11.7
Modest	361	53.6
Well-off	222	32.9
Wealthy	12	1.8

**Table 2:** Means, standard deviations, and correlations.

Variable	*M*	*SD*	1	2	3
1. Attachment anxiety	54.30	18.24	-		
2. Attachment avoidance	43.67	13.65	0.348 **	-	
3. Internalized homonegativity	24.58	8.63	0.224 **	0.256 **	-
4. Dyadic adjustment	144.17	15.97	−0.363 **	−0.525 **	−0.210 **

*Note. M* and *SD* are used to represent mean and standard deviation, respectively. ** indicates *p* < 0.01.

**Table 3:** Table of comparisons between women and men for attachment, internalized homonegativity, and dyadic adjustment dimensions.

Variable	Gender		t	df	*p*
	**Men** **M (SD)**	**Women** **M (SD)**			
Attachment anxiety	54.65 (18.10)	54.11 (18.33)	0.36	489.51	0.716
Attachment avoidance	45.80 (14.15)	42.51 (13.25)	2.95	457.82	0.003
Internalized homonegativity	25.87 (9.63)	23.89 (7.96)	2.71	412.38	0.007
Dyadic adjustment	143.96 (15.64)	144.28 (16.17)	−0.25	498.04	0.804

## Data Availability

The data presented in this study are available on request from the corresponding author for privacy reasons.

## Supplementary Materials

The following supporting information can be downloaded at: https://www.mdpi.com/article/10.3390/bs15020205/s1, Figure S1: Distributions of the estimates of the regression parameters of the multiple linear regression model calculated through the bootstrap method (1000 iterations) using the R Package Simpleboot (Peng, 2024).

## References

[B1-behavsci-15-00205] Acitelli L. A., Forgas J. P., Fitness J. (2008). Knowing when to shut up: Do relationship reflections help or hurt relationship satisfaction?. Social relationships: Cognitive, affective, and motivational processes.

[B2-behavsci-15-00205] Baiocco R., Scandurra C., Rosati F., Pistella J., Ioverno S., Bochicchio V., Wang H. C., Chang T. S. (2023). Minority stress, resilience, and health in Italian and Taiwanese LGB+ people: A cross-cultural comparison. Current Psychology.

[B3-behavsci-15-00205] Balsam K. F., Szymanski D. M. (2005). Relationship quality and domestic violence in women’s same-sex relationships: The role of minority stress. Psychology of Women Quarterly.

[B4-behavsci-15-00205] Bar-Shachar Y., Lopata S., Bar-Kalifa E. (2022). Relationship satisfaction during COVID-19: The role of partners’ perceived support and attachment. Family Relations.

[B5-behavsci-15-00205] Bowlby J. (1969). Attachment and loss, vol. 1: Attachment.

[B6-behavsci-15-00205] Bowlby J. (1973). Attachment and loss, vol. 2: Separation.

[B7-behavsci-15-00205] Bowlby J. (1980). Attachment and loss, vol. 3: Loss, sadness and depression.

[B8-behavsci-15-00205] Bradford A. B., Drean L., Sandberg J. G., Johnson L. N. (2020). They may disapprove, but i still love you: Attachment behaviors moderate the effect of social disapproval on marital relationship quality. Family Process.

[B9-behavsci-15-00205] Bresin K., Nicholas J. K., Cowand A. L., Alacha H. F., Rodriguez A. M., Parrott D. J. (2023). The effects of sexual and gender minority stress on relationship functioning: A meta-analysis. Personal Relationships.

[B10-behavsci-15-00205] Brown L. S. (1994). Subversive dialogues.

[B11-behavsci-15-00205] Busonera A., Martini P. S., Zavattini G. C., Santona A. (2014). Psychometric Properties of an Italian Version of the Experiences in Close Relationships-Revised (ECR-R) Scale. Psychological Reports.

[B12-behavsci-15-00205] Cataudella S., Rogier G., Beomonte Zobel S., Velotti P. (2023). The Relation of Anxiety and Avoidance Dimensions of Attachment to Intimate Partner Violence: A Meta-Analysis About Victims. Trauma, Violence & Abuse.

[B13-behavsci-15-00205] Delatorre M. Z., Wagner A. (2020). Marital Quality Assessment: Reviewing the Concept, Instruments, and Methods. Marriage & Family Review.

[B14-behavsci-15-00205] Doyle D. M., Molix L. (2015). Social stigma and sexual minorities’ romantic relationship functioning: A meta-analytic review. Personality & Social Psychology Bulletin.

[B15-behavsci-15-00205] Dürrbaum T., Sattler F. A. (2020). Minority stress and mental health in lesbian, gay male, and bisexual youths: A meta-analysis. Journal of LGBT Youth.

[B16-behavsci-15-00205] Farooqi S. R. (2014). The construct of relationship quality. Journal of Relationships Research.

[B17-behavsci-15-00205] Feinstein B. A., McConnell E., Dyar C., Mustanski B., Newcomb M. E. (2018). Minority stress and relationship functioning among young male same-sex couples: An examination of actor-partner interdependence models. Journal of Consulting and Clinical Psychology.

[B18-behavsci-15-00205] Fraley R. C., Waller N. G., Brennan K. A. (2000). An item response theory analysis of self-report measures of adult attachment. Journal of Personality and Social Psychology 78.

[B19-behavsci-15-00205] Frost D. M., Meyer I. H. (2009). Internalized homophobia and relationship quality among lesbians, gay men, and bisexuals. Journal of Counseling Psychology.

[B20-behavsci-15-00205] Frost D. M., Meyer I. H. (2023). Minority stress theory: Application, critique, and continued relevance. Current Opinion in Psychology.

[B21-behavsci-15-00205] Garbarini C. (2011). Adjustment di coppia: Confronto tra coppie senza figli, coppie con figli gemelli e coppie con figli mononati. International Journal of Developmental and Educational Psychology Revista INFAD de Psicología.

[B22-behavsci-15-00205] Garbarini C., Gerino E., Marino E., Rollé L., Brustia P. (2014). Psychometrical Properties of the Dyadic Adjustment Scale for Measurement of Marital Quality with Italian Couples. Procedia-Social and Behavioral Sciences.

[B23-behavsci-15-00205] Gentili P., Contreras L., Cassaniti M., D’Arista F. (2002). A measurement of dyadic adjustment: The Dyadic Adjustment Scale. Minerva Psichiatrica.

[B24-behavsci-15-00205] Hazan C., Shaver P. (1987). Romantic love conceptualized as an attachment process. Journal of Personality and Social Psychology.

[B25-behavsci-15-00205] Heene E. L., Buysse A., Van Oost P. (2005). Indirect pathways between depressive symptoms and marital distress: The role of conflict communication, attributions, and attachment style. Family Process.

[B26-behavsci-15-00205] Hoy-Ellis C. P. (2023). Minority stress and mental health: A review of the literature. Journal of Homosexuality.

[B27-behavsci-15-00205] Itzhaky L., Stein J. Y., Levin Y., Solomon Z. (2017). Posttraumatic stress symptoms and marital adjustment among Israeli combat veterans: The role of loneliness and attachment. Psychological Trauma: Theory, Research, Practice and Policy.

[B28-behavsci-15-00205] Kamen C., Burns M., Beach S. R. (2011). Minority stress in same-sex male relationships: When does it impact relationship satisfaction?. Journal of Homosexuality.

[B29-behavsci-15-00205] Kinsey A. C., Pomeroy W. B., Martin C. E. (1948). Sexual behavior in the human male.

[B30-behavsci-15-00205] Lancaster M., Seibert G. S., Cooper A. N., May R. W., Fincham F. (2020). Relationship quality in the context of cyber dating abuse: The role of attachment. Journal of Family Issues.

[B31-behavsci-15-00205] Lewis R. J., Milletich R. J., Derlega V. J., Padilla M. A. (2014). Sexual minority stressors and psychological aggression in lesbian women’s intimate relationships: The mediating roles of rumination and relationship satisfaction. Sychology of Women Quarterly.

[B32-behavsci-15-00205] Li X., Cao H., Zhou N., Mills-Koonce R. (2021). Internalized homophobia and relationship quality among same-sex couples: The mediating role of intimate partner violence. Journal of homosexuality.

[B33-behavsci-15-00205] Lingiardi V., Baiocco R., Nardelli N. (2012). Measure of internalized sexual stigma for lesbians and gay men: A new scale. Journal of Homosexuality.

[B34-behavsci-15-00205] Mann A. M., Naugle A. E., Lieberman E. (2022). Experiential avoidance and emotion dysregulation as mediators in the LGBTQ minority stress model. Archive of Sexual Behaviors.

[B35-behavsci-15-00205] Meyer I. H. (1995). Minority stress and mental health in gay men. Journal of Health & Social Behavior.

[B36-behavsci-15-00205] Meyer I. H. (2003). Prejudice, social stress, and mental health in lesbian, gay and bisexual populations: Conceptual issues and research evidence. Psychological Bulletin.

[B37-behavsci-15-00205] Meyer I. H., Dean L., Herek G. (1998). Internalized homophobia, intimacy and sexual behaviour among gay and bisexual men. Stigma and sexual orientation.

[B38-behavsci-15-00205] Meyer I. H., Frost D. M., Patterson C., D’Augelli A. R. (2013). Minority stress and the health of sexual minorities. Handbook of psychology and sexual orientation.

[B39-behavsci-15-00205] Mikulincer M., Shaver P. R. (2005). Attachment theory and emotions in close relationships: Exploring the attachment-related dynamics of emotional reactions to relational events. Personal Relationships.

[B40-behavsci-15-00205] Mikulincer M., Shaver P. R. (2007a). Attachment in adulthood: Structure, dynamics, and change.

[B41-behavsci-15-00205] Mikulincer M., Shaver P. R. (2007b). Boosting attachment security to promote mental health, prosocial values, and inter-group tolerance. Psychological Inquiry.

[B42-behavsci-15-00205] Mikulincer M., Shaver P. R., Cassidy J., Shaver P. R. (2008). Adult attachment and affect regulation. Handbook of attachment: Theory, research, and clinical applications.

[B43-behavsci-15-00205] Mikulincer M., Shaver P. R. (2014). An attachment perspective on loneliness. The handbook of solitude: Psychological perspectives on social isolation, social withdrawal, and being alone.

[B44-behavsci-15-00205] Mikulincer M., Shaver P. R. (2016). Attachment in adulthood: Structure, dynamics, and change.

[B45-behavsci-15-00205] Mikulincer M., Shaver P. R. (2019). Attachment orientations and emotion regulation. Current Opinion in Psychology.

[B46-behavsci-15-00205] Mikulincer M., Shaver P. R., Pereg D. (2003). Attachment theory and affect regulation: The dynamics, development, and cognitive consequences of attachment-related strategies. Motivation and Emotion.

[B47-behavsci-15-00205] Mohr J., Cassidy J., Shaver P. R. (2008). Same-sex romantic attachment. Handbook of attachment theory and research.

[B48-behavsci-15-00205] Mohr J. J., Selterman D., Fassinger R. E. (2013). Romantic attachment and relationship functioning in same-sex couples. Journal of Counseling Psychology.

[B49-behavsci-15-00205] Morry M. M., Reich T., Kito M. (2010). How do I see you relative to myself? Relationship quality as a predictor of self-and partner-enhancement within cross-sex friendships, dating relationships, and marriages. The Journal of Social Psychology.

[B50-behavsci-15-00205] Newcomb M. E., Mustanski B. (2010). Internalized homophobia and internalizing mental health problems: A meta-analytic review. Clinical Psychology Review.

[B51-behavsci-15-00205] Pachankis J. E., Rendina H. J., Restar A., Ventuneac A., Grov C., Parsons J. T. (2015). A minority stress-emotion regulation model of sexual compulsivity among highly sexually active gay and bisexual men. Health Psychology.

[B52-behavsci-15-00205] Peng R. D. (2024). simpleboot: Simple bootstrap routines. R package version 1.1-8.

[B53-behavsci-15-00205] R Development Core Team (2021). R: A language and environment for statistical computing.

[B54-behavsci-15-00205] Rendina H. J., Gamarel K. E., Pachankis J. E., Ventuneac A., Grov C., Parsons J. T. (2017). Extending the minority stress model to incorporate HIV-positive gay and bisexual men’s experiences: A longitudinal examination of mental health and sexual risk behavior. Annals of Behavioral Medicine: A Publication of the Society of Behavioral Medicine.

[B55-behavsci-15-00205] Rosenthal L., Starks T. J. (2015). Relationship stigma and relationship outcomes in interracial and same-sex relationships: Examination of sources and buffers. Journal of Family Psychology.

[B56-behavsci-15-00205] Rostosky S. S., Riggle E. D. B. (2017). Same-sex relationships and minority stress. Current Opinion in Psychology.

[B57-behavsci-15-00205] Santoniccolo F., Trombetta T., Rollè L. (2021). The help-seeking process in same-sex intimate partner violence: A systematic review. Sexuality Research and Social Policy.

[B58-behavsci-15-00205] Scott S. B., Parsons A. M., Do Q. A., Knopp K., Rhoades G. K. (2023). Actor-partner effects of sexual minority stress on relationship quality in female same-gender couples. Couple and Family Psychology: Research and Practice.

[B59-behavsci-15-00205] Shaver P. R., Mikulincer M. (2002). Attachment-related psychodynamics. Attachment & Human Development.

[B60-behavsci-15-00205] Shaver P. R., Mikulincer M., Gross J. T., Stern J. A., Cassidy J. A., Cassidy J., Shaver P. R. (2016). A Lifespan perspective on attachment and care for others: Empathy, altruism, and prosocial behavior. Handbook of attachment: Theory, research, and clinical applications.

[B61-behavsci-15-00205] Simpson J. A. (1990). Influence of attachment styles on romantic relationships. Journal of Personality and Social Psychology.

[B62-behavsci-15-00205] Sochos A., Yahya F. (2015). Attachment style and relationship difficulties in parents of children with ADHD. Journal of Child and Family Studies.

[B63-behavsci-15-00205] Sommantico M., Donizzetti A. R., Parrello S., De Rosa B. (2019). Gay and lesbian couples’ relationship quality: Italian validation of the Gay and Lesbian Relationship Satisfaction Scale (GLRSS). Journal of Gay & Lesbian Mental Health.

[B64-behavsci-15-00205] Song C., Buysse A., Zhang W.-H., Dewaele A. (2023). Minority stress and relationship satisfaction in same-sex couples: A meta-analysis. Family Relations: An Interdisciplinary Journal of Applied Family Studies.

[B65-behavsci-15-00205] Song C., Buysse A., Zhang W.-H., Lu C., Zhao M., Dewaele A. (2022). Coping with minority stress in romantic relationships among lesbian, gay and bisexual people. Current Psychology: A Journal for Diverse Perspectives on Diverse Psychological Issues.

[B66-behavsci-15-00205] Spanier G. B. (1976). Measuring dyadic adjustment: New scales for assessing the quality of marriage and similar dyads. Journal of Marriage and the Family.

[B67-behavsci-15-00205] Spanier G. B., Cole C. L. (1975). Mate swapping: Perceptions, value orientations, and participation in a midwestern community. Archives of Sexual Behavior.

[B68-behavsci-15-00205] Sullivan T. J., Feinstein B. A., Marshall A. D., Mustanski B. (2017). Trauma exposure, discrimination, and romantic relationship functioning: A longitudinal investigation among LGB young adults. Psychology of Sexual Orientation and Gender Diversity.

[B69-behavsci-15-00205] Szymanski D. M., Dunn T. L., Ikizler A. S. (2014). Multiple minority stressors and psychological distress among sexual minority women: The roles of rumination and maladaptive coping. Psychology of Sexual Orientation and Gender Diversity.

[B70-behavsci-15-00205] Szymanski D. M., Hilton A. N. (2013). Fear of intimacy as a mediator of the internalized heterosexism-relationship quality link among men in same-sex relationships. Contemporary Family Therapy: An International Journal.

[B71-behavsci-15-00205] Tognasso G., Trombetta T., Gorla L., Ramon S., Santona A., Rollè L. (2022). Romantic attachment, internalized homonegativity, and same-sex intimate partner violence perpetration among lesbian women in Italy. Frontiers in Psychology.

[B72-behavsci-15-00205] Trombetta T., Balocco V., Santoniccolo F., Paradiso M. N., Rollè L. (2023). Internalized homonegativity, emotion dysregulation, and isolating behaviors perpetration among gay and lesbian couples. International Journal of Environmental Research and Public Health.

[B73-behavsci-15-00205] Trombetta T., Paradiso M. N., Santoniccolo F., Rollè L. (2024). The role of attachment anxiety and avoidance in predicting proximal minority stressors among gay and lesbian people in Italy. International Journal of Environmental Research and Public Health.

[B74-behavsci-15-00205] Trombetta T., Rollè L. (2022). Intimate partner violence perpetration among sexual minority people and associated factors: A systematic review of quantitative studies. Sexuality Research & Social Policy.

[B75-behavsci-15-00205] Trombetta T., Rollè L. (2023). Internalized homonegativity, emotion dysregulation, and physical same-sex intimate partner violence perpetration: A psychological mediation framework-based model. Journal of Interpersonal Violence.

[B76-behavsci-15-00205] Vigl J., Strauss H., Talamini F., Zentner M. (2022). Relationship satisfaction in the early stages of the COVID-19 pandemic: A cross-national examination of situational, dispositional, and relationship factors. PLoS ONE.

[B77-behavsci-15-00205] Zakalik R. A., Wei M. (2006). Adult attachment, perceived discrimination based on sexual orientation, and depression in gay males: Examining the mediation and moderation effects. Journal of Counseling Psychology.

[B78-behavsci-15-00205] Zevy L., Cavallaro S. A., Boston Lesbian Psychologies Collective (1987). Invisibility, fantasy, and intimacy: Princess charming is not a prince. Lesbian psychologies: Explorations and challenges.

[B79-behavsci-15-00205] Zhang S., Li T. (2021). Attachment style moderates the relationship between solitude and marital satisfaction: The cross-partner effects. Family Relations: An Interdisciplinary Journal of Applied Family Studies.

[B80-behavsci-15-00205] Zhang X., Li J., Xie F., Chen X., Xu W., Hudson N. W. (2022). The relationship between adult attachment and mental health: A meta-analysis. Journal of Personality & Social Psychology.

[B81-behavsci-15-00205] Zheng L., Luo Y., Chen X. (2020). Different effects of attachment anxiety and attachment avoidance on depressive symptoms: A meta-analysis. Journal of Social & Personal Relationships.

