# Supplementary material for: Untangling Relational Ties: How Internalized Homonegativity and Adult Attachment Shape Relationship Quality in Lesbian and Gay Couples
Source: Behav Sci (Basel). 2025 Feb 13;15(2):205. doi: 10.3390/bs15020205 (PMC11851490; doi:10.3390/bs15020205)

Supplementary Materials

Distributions of the estimates of the regression parameters of the multiple linear regression model calculated through the bootstrap method (1000 iterations) using the R Package *Simpleboot* (Peng, 2024).

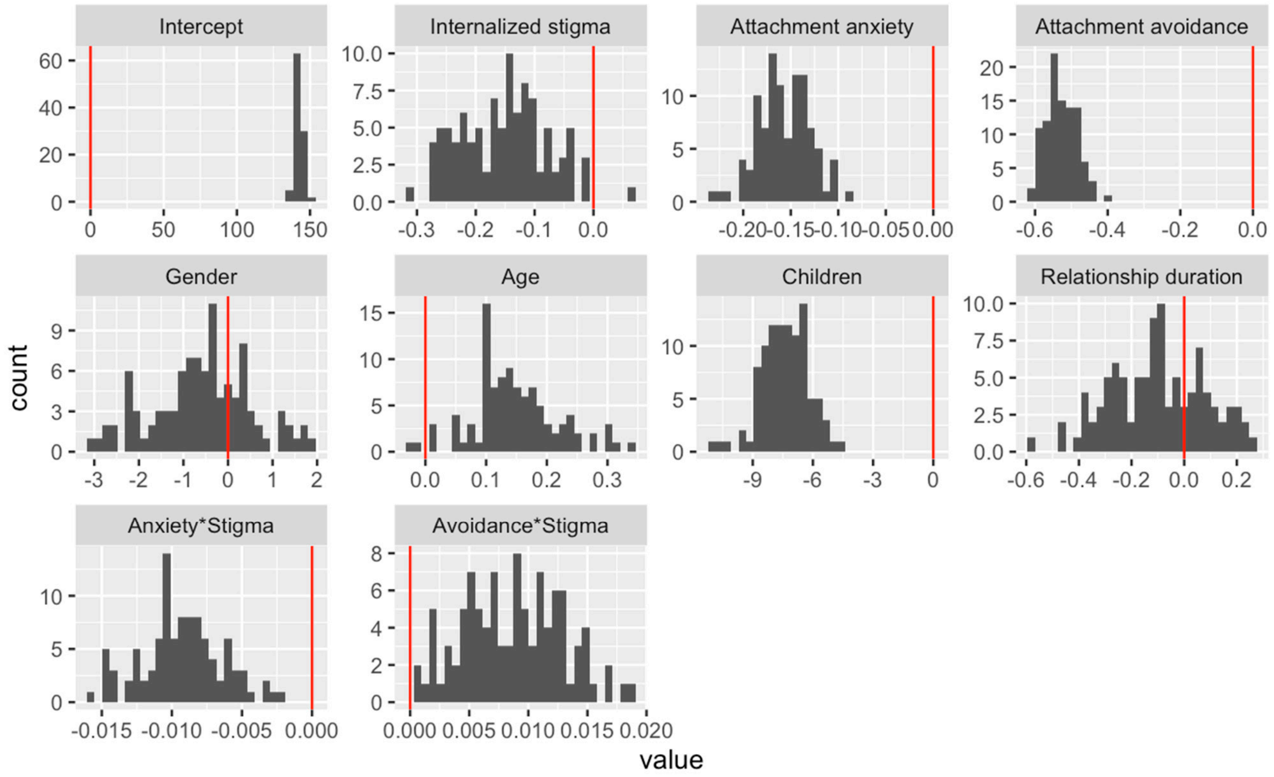

Supplement: Supplementary file 1 [file behavsci-15-00205-s001.zip › behavsci-3450510-supplementary.pdf]
